# Egr2 and 3 maintain anti-tumour responses of exhausted tumour infiltrating CD8 + T cells

**DOI:** 10.1007/s00262-022-03319-w

**Published:** 2022-11-07

**Authors:** Alistair L. J. Symonds, Tizong Miao, Zabreen Busharat, Suling Li, Ping Wang

**Affiliations:** 1 The Blizard Institute, Barts and The London School of Medicine and Dentistry, Queen Mary University of London, 4 Newark Street, London, E1 2AT UK; 2 Bioscience, Brunel University, Kingston Lane, London, UB8 3PH UK

**Keywords:** Egr2, Egr3, Tumour infiltrating lymphocytes, Anti-PD-1

## Abstract

Although T cells can develop into an exhausted state in the tumour microenvironment, tumour infiltrating T cells (TILs) are important to control tumour growth. By analysing single cell RNA-sequencing data from human tumours, we found that the transcription factors Early Growth Response 2 (EGR2) and 3 were highly induced in TILs, but not peripheral CD8 + T cells, in multiple patient cohorts. We found that deficiency of Egr2 and 3 in T cells resulted in enhanced tumour growth and fewer TILs in mouse models. Egr2 is highly expressed together with checkpoint molecules in a proportion of CD8 + TILs and Egr2high cells exhibit better survival and proliferation than Egr2^-/-^Egr3^-/-^ and Egr2low TILs. Anti-PD-1 treatment increases Egr2 expression in CD8 + TILs and reduces tumour growth, while anti-PD-1 efficacy is abrogated in the absence of Egr2 and 3. Thus, Egr2 and 3 are important for maintaining anti-tumour responses of exhausted CD8 + TILs.

**Supplementary Information:**

The online version contains supplementary material available at 10.1007/s00262-022-03319-w.

## Introduction

Immune surveillance mediated by T cells is important to control tumour growth [1]. However, the function of tumour specific T cells can be progressively modulated by factors present in the tumour microenvironment (TME), such as persistent tumour antigens and inflammatory factors [2]. CD3 + CD8 + Tumour infiltrating lymphocytes (TILs) frequently display an exhausted state with high expression of immune checkpoint regulators, dysregulated metabolic pathways and impaired proliferation and effector function [2]. However, although exhausted T cells are dysfunctional compared to effector cells, they can be reinvigorated by blockade of immune checkpoint molecules [3], implying that they may still retain anti-tumour immune function. Transcriptomic and epigenetic analysis of exhausted CD8 T cells revealed a heterogeneous phenotype and function of different exhausted cell subsets and a number of transcription factors such as TCF-1, Tox and T-bet have been found to modulate their effector functions [4]. The transcription factors Egr2 and/or 3 have also been detected in TILs from both tumour models and human solid tumours [5–8]. Egr2 and 3 have dynamic function in effector T cells [9–12]. They are essential for maintaining optimal responses of T cells against viral infection by promoting proliferation but controlling excessive inflammatory effector function [10, 11]. Egr2/3 also have an important function in the homeostasis of memory phenotype T cells [13–15] as they are essential to maintain the homeostatic turnover of these cells while controlling autoimmune inflammatory functions [13]. Since Egr2/3 are negative regulators of inflammatory functions in viral specific CD8 T cells [11, 12], they may play a role in the dysfunction of exhausted TILs, which has been suggested by the co-expression of Egr2 and checkpoint regulators such as Lag3 [5, 6]. Alternatively, the function of Egr2 and 3 in regulating proliferation and survival of effector T cells [11, 12] may be important for TIL function.

In this study, we discovered that, in contrast to TCF-1, Egr2 and 3 were specifically induced in a proportion of CD8 + TILs in multiple human tumours. Using mouse models, we found that Egr2/3 were co-expressed with multiple checkpoint regulators in a proportion of TILs and deficiency of Egr2/3 in T cells resulted in aggressive tumour growth. In the absence of Egr2/3, the number of TILs is reduced significantly in tumours and TIL proliferative responses are impaired. However, the effector function of Egr2/3 deficient T cells was not impaired indicating that Egr2/3 do not induce exhaustion, but have an independent and intrinsic role in maintaining the function of exhausted TILs.

## Results

### Egr2 and/or 3 are highly induced in a subset of CD8 + TILs in human tumours

**Fig. 1:**
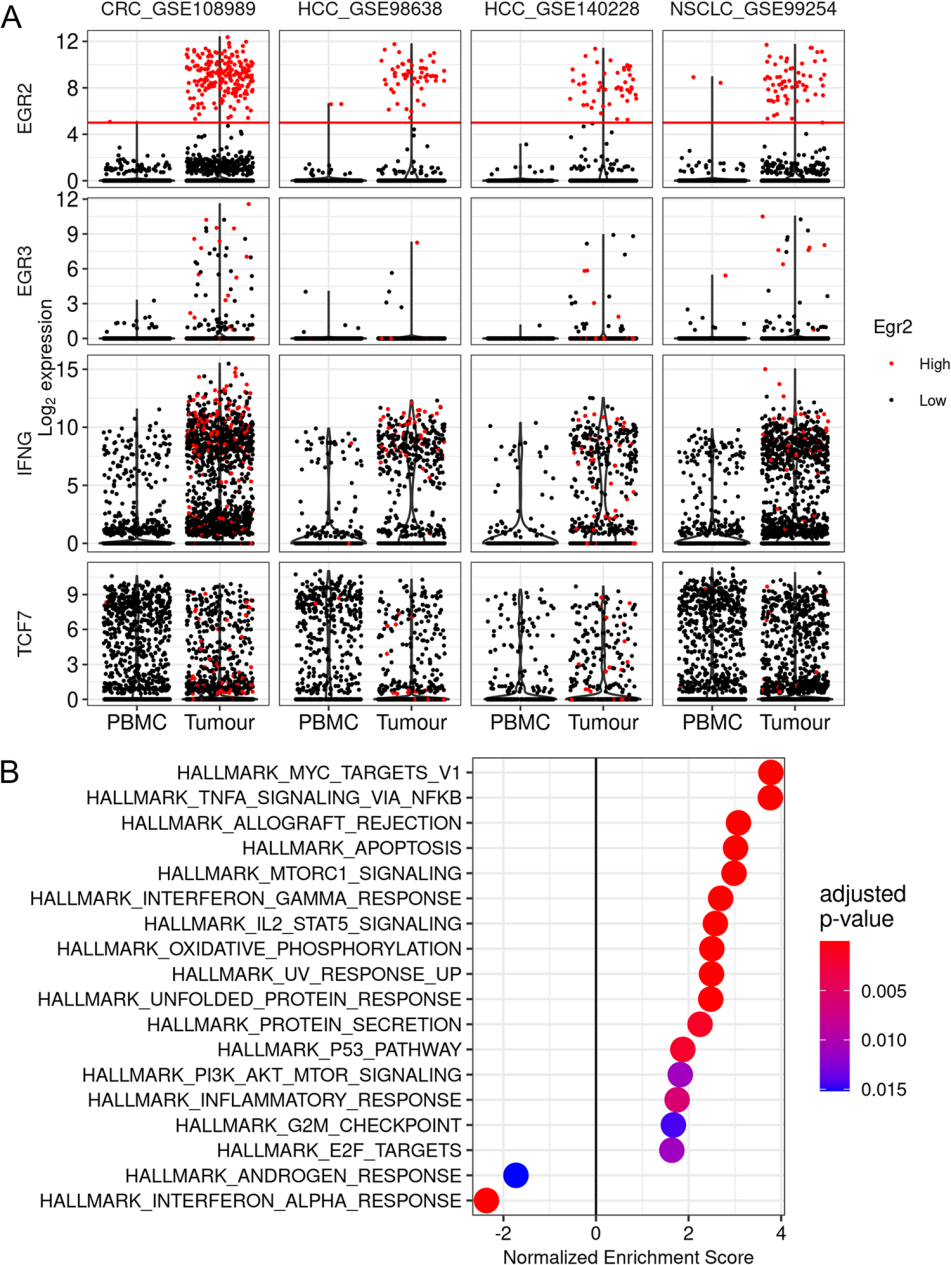
Egr2 and 3 are expressed in CD8 + T cells from human tumours. Analysis of single cell RNA sequencing data from CD8 T cells from human tumours from four reported studies (CRC, colorectal cancer; HCC, hepatocellular carcinoma; NSCLC, Non-small cell lung cancer). **A**. Normalised expression of EGR2, EGR3, IFNG and TCF7. Cells are coloured by EGR2 expression level, with EGR2 high cells (defined as log2 expression greater than 5, as shown by the red line) coloured red and EGR2 low cells coloured black. **B**. Gene set enrichment analysis of pseudobulk data generated from EGR2high and EGR2low cells from tumour infiltrating CD8 T cells from dataset CRC_GSE108989. For each patient, two pseudobulk samples were generated from their EGR2 high cells and EGR2low cells, respectively. A LRT test was performed using DESeq2 and used in pre-ranked GSEA using the Broad Institute Hallmark genesets [37]. Normalised enrichment is shown, with genesets enriched in EGR2high cells having a positive score and genesets enriched in EGR2low cells having a negative score

It has been reported that exhausted TILs are diverse in both phenotypes and function [7, 16–21]. TCF-1, encoded by *TCF7*, has been suggested to maintain the function of a proportion of CD8 + TILs that are at an early stage of exhaustion [22, 23]. However, analysis of single cell RNA-sequencing data from cohorts of colorectal, liver and lung cancer patients [18–21] showed that the percentages of TCF7 expressing CD8 + T cells are not increased in TILs compared to peripheral blood CD8 T cells (Fig. 1A), indicating that TCF7 is not induced specifically in the tumour microenvironment. In contrast, the transcription factor Egr2 was highly induced in a subset of CD8 + TILs, but not in CD8 + T cells from peripheral blood (Fig. 1A). Fewer cells expressed Egr3 but it was similarly preferentially expressed in TILs (Fig. 1A). The percentage of Egr2 positive TILs varied greatly in different tumours with ~ 30% in TILs from colorectal cancer, ~ 15% in those from liver cancer and ~ 9% in TILs from lung cancer (Fig. 1A, Supplementary Table 1). The percentage of Egr2 positive CD8 + TILs also varied greatly among patients with the same cancer, ranging from 10% to more than 40% in individual colorectal cancer patients (Supplementary Table 2). To assess the functional pathways potentially regulated by Egr2, we performed Gene set enrichment analysis (GSEA) on pseudobulk samples formed from Egr2 high and Egr2 low cells from one data set from a cohort of colorectal cancer patients [18]. Although Egr2high CD8 + TILs express high levels of checkpoint molecules, suggesting that they are exhausted [5, 7], the genesets that were enriched in Egr2high compared to Egr2 low CD8 + TILs were involved in pathways driving T cell activation and effector function (Fig. 1B). These results suggest that Egr2high CD8 + TILs may have better function in anti-tumour immune responses than their Egr2 low counterparts.

### Egr2 and 3 are important for TIL mediated immune responses against tumours

**Fig. 2:**
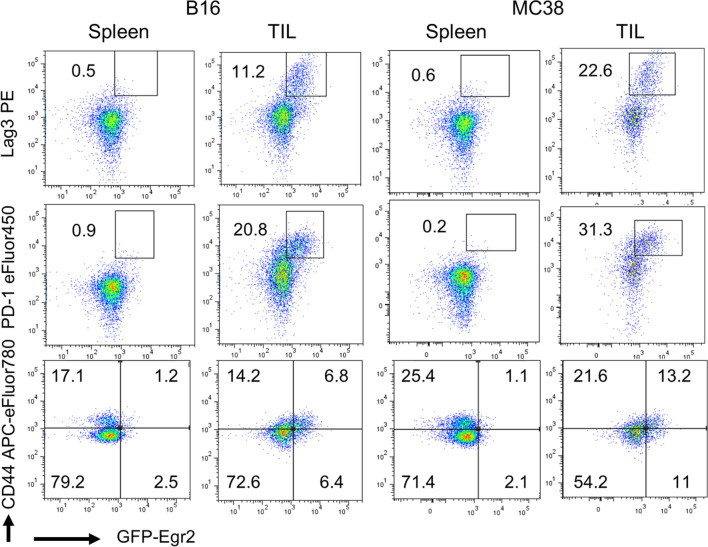
Egr2 is induced in CD8 + TILs, but not peripheral CD8 + T cells in mouse tumour models. MC38 and B16 tumour models were established in Kin (GFP-Egr2) mice. The expression of Egr2, PD-1, Lag3 and the memory phenotype marker CD44 was analysed on CD8 + T cells from spleens and tumours 14 days after tumour cell inoculation. The results are representative of groups of five mice from two independent experiments

**Fig. 3:**
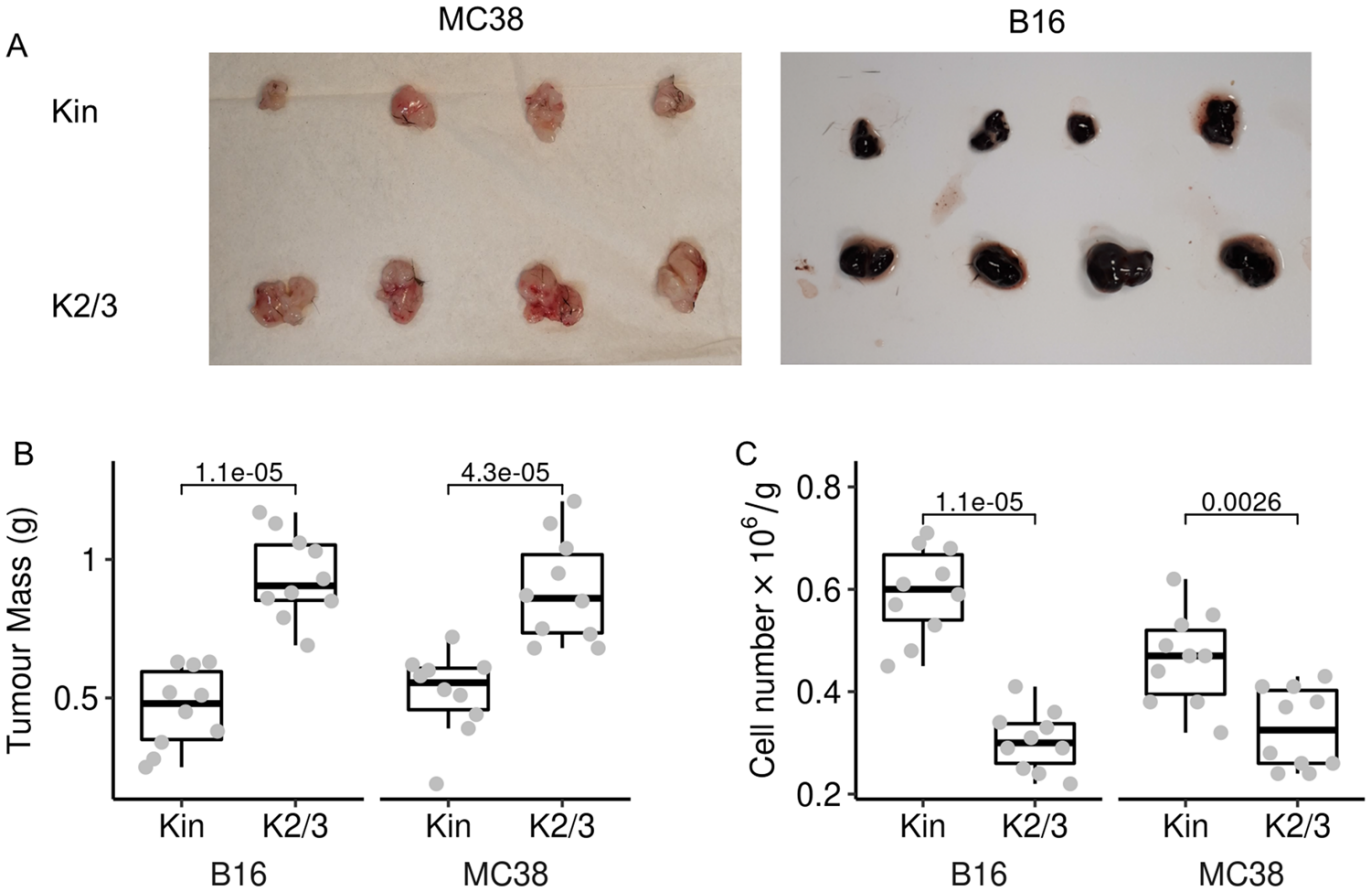
Deficiency of Egr2/3 in T cells reduces TIL cell numbers and increases tumour growth. Tumours were established in Kin (GFP-Egr2) and K2/3 (CD2-Egr2/3^-/-^) mice and analysed 14 days after injection. **A** and **B**. Tumours were isolated and compared. **C**. CD8 + TILs were isolated from the tumours and numbers were compared between Kin and K2/3 groups. Data in **A** are representative of 3 independent experiments. In **B** and **C**, the median, upper and lower quartiles from groups of 10 mice are shown and data were analysed with two-tailed Mann–Whitney tests

Egr2 and 3 have overlapping functions in T cells in mice [9–11, 13, 14]. To examine the impact of Egr2 and Egr3 in T cells on anti-tumour responses, the growth of two tumour models, B16 and MC38, was assessed in GFP-Egr2 knockin and CD2-Egr2/3^-/-^ mice. Consistent with the findings in human tumours (Fig. 1), Egr2 was detected in a proportion of CD8 + TILs, but not peripheral CD8 + T cells (Fig. 2, Supplementary Fig. 1). Deficiency of Egr2 and 3 in T cells resulted in excessive tumour growth (Fig. 3A, B). The number of CD8 + TILs per gram in both tumour models from CD2-Egr2/3^-/-^ mice were much less than GFP-Egr2 knockin mice (Fig. 3C). These results indicate that Egr2/3 are important for anti-tumour responses of CD8 + TILs.

### Egr2highCD8 + TILs express the highest levels of checkpoint molecules

**Fig. 4:**
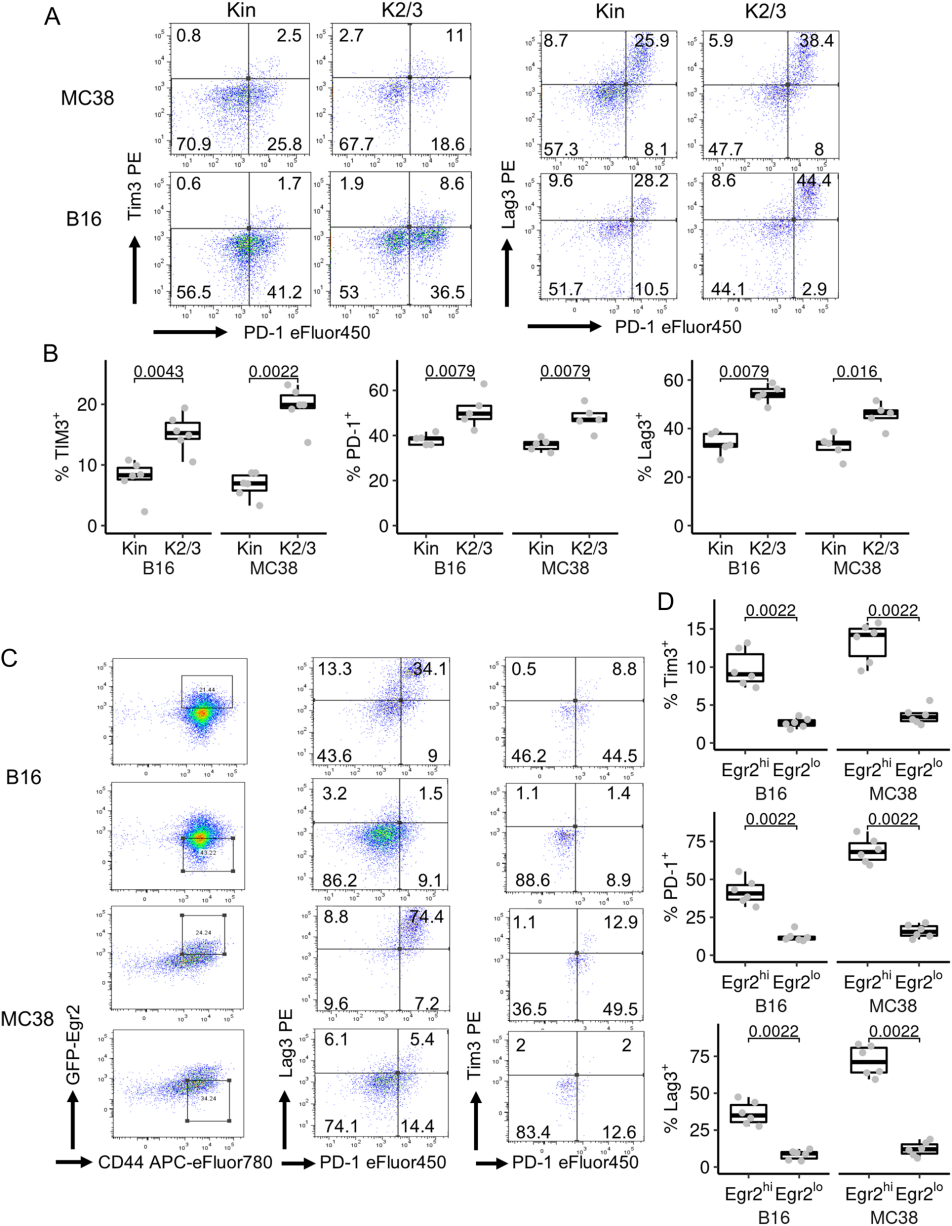
Checkpoint molecule expression by GFP-Egr2high, GFP-Egr2low and Egr2/3^-/-^ TILs. MC38 or B16 tumour cells were injected into Kin (GFP-Egr2) and K2/3 (CD2-Egr2/3^-/-^) mice and TILs were analysed 14 days later. **A** and **B**. CD8 + TILs from Kin and K2/3 mice were analysed for expression of the indicated markers. **C** and **D**. CD8 + TILs were isolated from Kin mice and Egr2high and low cells were gated for analysis of checkpoint molecules. Data in **A** and **C** are representative of 6 (**C** and **A**, PD-1 and TIM3) or 5 (**A**, PD-1 and Lag3) mice in each group and are representative of two independent experiments. In **B** and **D**, the median, upper and lower quartiles from groups of 5 (**B**, PD-1 and Lag3) or 6 (**D** and **B**, PD-1 and TIM3) mice are shown and data were analysed with two-tailed Mann–Whitney tests

Egr2 has been suggested to induce checkpoint molecules in TILs [5]. The percentages of CD8 + TILs from CD2-Egr2/3^-/-^ mice expressing Lag3, Tim3 and PD-1 were higher than those from GFP-Egr2 knockin mice (Fig. 4A, B), indicating that Egr2 and 3 are not required for expression of immune checkpoint molecules. However, analysis of Egr2high and Egr2low CD8 + TILs in GFP-Egr2 knockin mice revealed that Egr2high CD8 + TILs expressed much higher levels of checkpoint molecules than Egr2low CD8 + cells (Fig. 4C, D), consistent with the findings from Egr2high CD8 + TILs in human tumours [7]. The expression of checkpoint molecules in a large proportion of Egr2/3^-/-^ TILs may result from the previously reported homeostatic disorder in these mice, which leads to hyper activation and expression of checkpoint molecules as reported previously [9, 14]. Collectively, these data suggest that Egr2 and/or 3 are induced concomitantly with checkpoint molecules in exhausted T cells in the tumour microenvironment rather than driving checkpoint molecule expression.

### Egr2/3 are important for survival and expansion of exhausted TILs

**Fig. 5:**
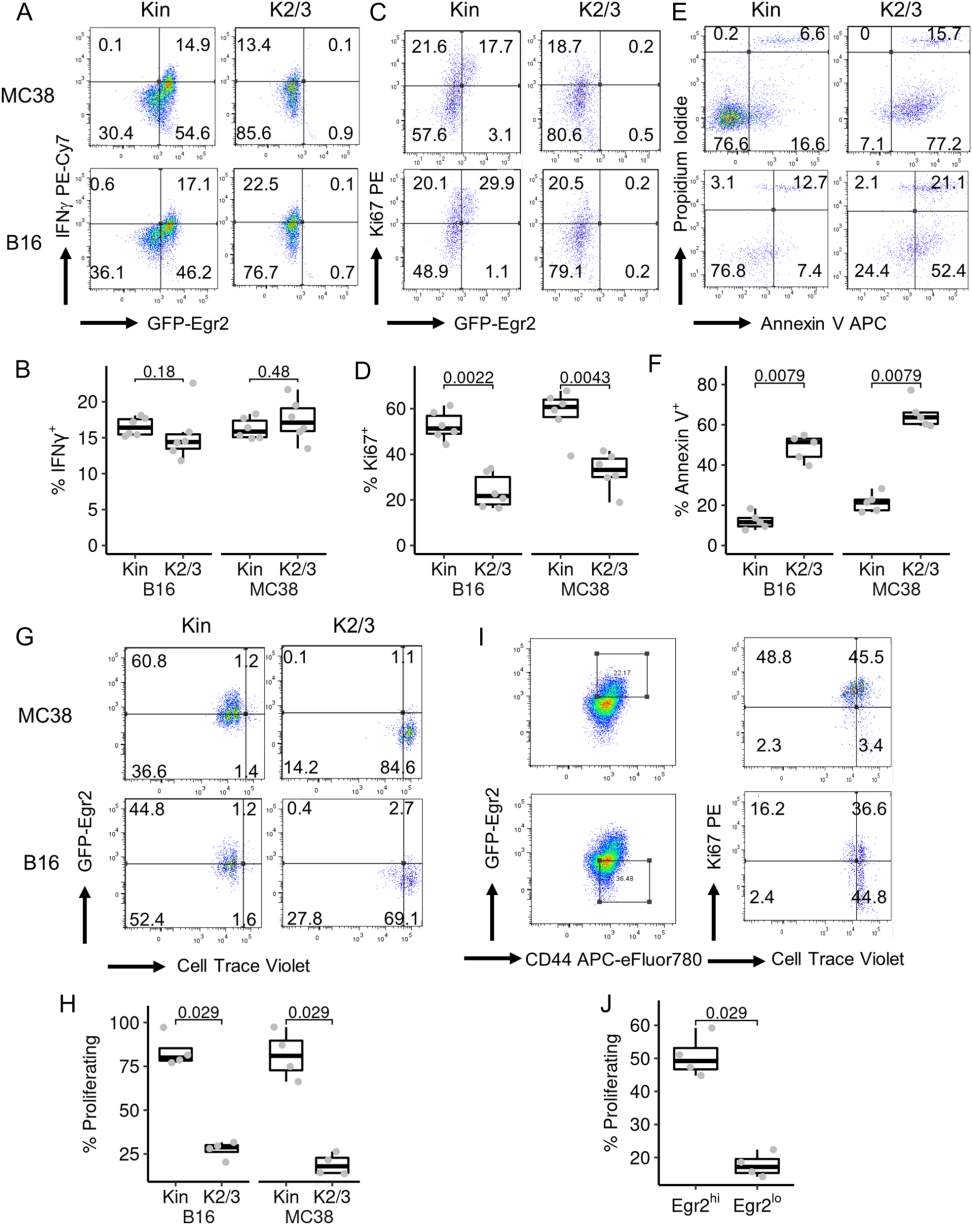
Deficiency of Egr2/3 in T cells impairs expansion and survival of CD8 + TIL, but has little effect on IFNγ production. TILs were isolated from MC38 or B16 tumours from Kin (GFP-Egr2) and K2/3 (CD2-Egr2/3^-/-^) mice 14 days after inoculation. **A–F**. IFNγ production (**A** and **B**), Ki67 expression (**C** and **D**) and Annexin V staining (**E** and **F**) were analysed. **G** and **H**. CD8 + TILs were labelled with Cell Trace Violet and stimulated for 72 h with anti-CD3 and anti-CD28 in vitro. After stimulation, proliferation was analysed by flow cytometry. **I** and **J**. CD8 + TILs from MC38 tumours from Kin mice were labelled with Cell Trace Violet before stimulation with anti-CD3 and anti-CD28 in vitro for 72 h. After stimulation, gated GFP-Egr2high and GFP-Egr2low cells were analysed for Ki67 expression and proliferation. Data in **A**, **C**, **E**, **G**, **I** are representative of 6 (**A**,** C**), 5 (**E**) or 4 (**G, I**) mice in each group and are representative of two independent experiments. In **B**, **D**, **F**, **H** and **J** the median, upper and lower quartiles from groups of 6 (**B** and **D**), 5 (**F**) or 4 (**H**, **J**) mice are shown and data were analysed with two-tailed Mann–Whitney tests

Exhausted T cells display impaired proliferation and effector function [4, 16]. Therefore, we assessed the roles of Egr2 and 3 in the function, proliferation and survival of CD8 + TILs. The percentages of IFNγ producing CD8 + TILs were similar in GFP-Egr2 knockin and CD2-Egr2/3^-/-^ mice; likewise the proportions of CD8 + TILs expressing TCF-1 were comparable in GFP-Egr2 knockin and CD2-Egr2/3^-/-^ mice (Fig. 5A, B, Supplementary Figs. 2 and 3). However, the proportion of CD8 T cells expressing Ki67, a proliferation marker, was much higher in tumours from GFP-Egr2 knockin mice than in CD2-Egr2/3^-/-^ mice (Fig. 5C, D). Moreover, the majority of Egr2/3^-/-^ CD8 + TILs were Annexin V + while TILs from GFP-Egr2 knockin mice were mostly Annexin V negative (Fig. 5E, F). In addition to signs of cell death, Egr2/3^-/-^ CD8 + TILs were severely impaired in proliferation in response to TCR stimulation in vitro (Fig. 5G, H). Interestingly, despite expression of high levels of immune checkpoint molecules (Fig. 4C, D), Egr2high CD8 + TILs showed superior proliferation to Egr2low CD8 TILs in response to TCR stimulation in vitro (Fig. 5I, J). These results suggest that Egr2/3 are important for the survival and expansion of TILs which is consistent with similar roles in effector T cells induced in response to viral infection [11].

### Impaired expression of genes involved in proliferation and metabolism in Egr2/3 deficient CD8 + TILs

**Fig. 6:**
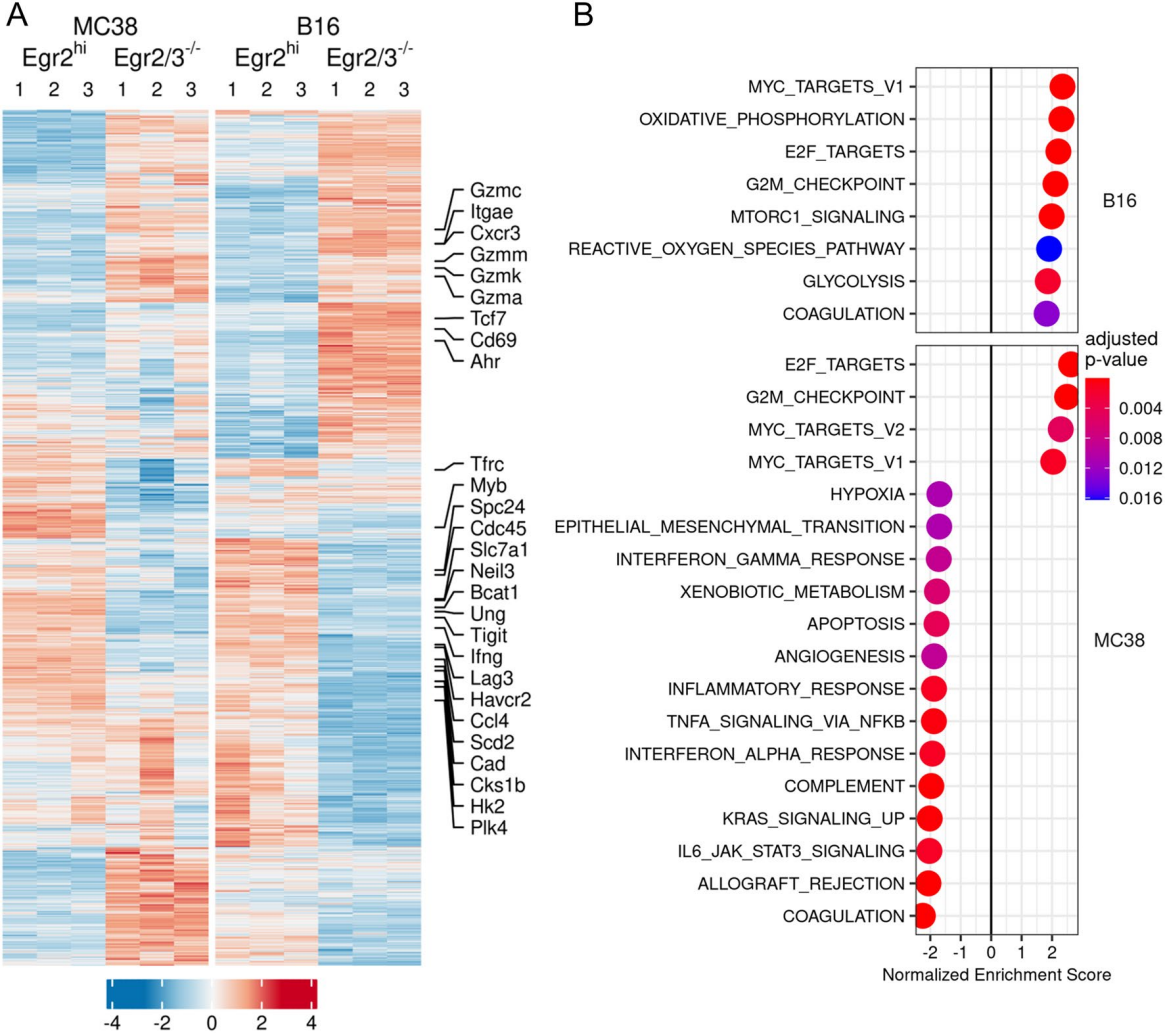
Defective expression of genes involved in proliferation and metabolism in Egr2/3^-/-^ CD8 + TILs. MC38 and B16 tumours were established in Kin (GFP-Egr2) and K2/3 (CD2-Egr2/3^-/-^) mice. 14 days later, GFP-Egr2high and Egr2/3^- /-^ TILs were isolated from Kin and K2/3 mice, respectively, and analysed by RNA-seq. A. Unsupervised hierarchical clustering of differentially expressed genes showing expression patterns in GFP-Egr2high and Egr2/3^-/-^ TILs. Selected genes relevant to TIL function are indicated. B. GSEA of Hallmark gene sets [37] for GFP-Egr2high vs Egr2/3^-/-^ TILs. Normalised enrichment is shown, with genesets enriched in GFP-Egr2high TILs having a positive score and genesets enriched in Egr2/3^-/-^ TILs having a negative score. The RNA-seq data are from three biological replicates, each with cells pooled from 10 mice, for each group

To assess the impact of Egr2 on TIL gene expression programmes, we isolated Egr2high CD8 + TILs and Egr2/3^-/-^ CD8 + TILs from Kin and K2/3 mice, respectively, and analysed them by RNAseq. This revealed that checkpoint molecules Lag3, Tigit and Havcr2 were decreased in Egr2/3^-/-^ CD8 + TILs, while tissue residency markers Cd69 and Itgae were increased (Fig. 6A, Supplementary Data files 1 and 2). Tcf7, a transcription factor expressed in early stage exhausted T cells [22, 23], was also increased in Egr2/3^-/-^ CD8 + TILs in B16 tumours and unchanged in MC38 while genes involved in cell growth (Myb, Plk4, Cdc45, Cks1b, Spc24), DNA repair (Ung1, Neil3) and metabolism (Hk2, Cad, Bcat1, Scd2, Slc7a1, Tfrc) were decreased in Egr2/3^-/-^ CD8 + TILs in both models (Fig. 6A, Supplementary Data files 1 and 2). Genes associated with effector function of CD8 T cells showed a heterogeneous pattern, with many genes, such as Gzmk, Gzma, Gzmm, Gzmc, Cxcr3, Ahr, increased in Egr2/3^-/-^ CD8 + TILs, while others such as Ccl4 and Ifng were decreased in Egr2/3^-/-^ CD8 + TILs (Fig. 6A, Supplementary Data files 1 and 2). Gene Set Enrichment Analysis confirmed that pathways associated with cell growth, such as G2M checkpoint, Myc and E2F targets, were reduced in Egr2/3^-/-^ TILs, while some inflammatory pathways were increased in the MC38 model (Fig. 6B). Importantly, some of these pathways regulated by Egr2 and 3 were also enriched in Egr2high CD8 + TILs from human tumours (Fig. 1B). These results suggest a mechanism by which Egr2/3 maintain the adaptive immune responses of exhausted CD8 + TILs by promoting metabolism and expansion.

### Deficiency of Egr2/3 impairs anti-PD-1 blockade mediated anti-tumour responses of TILs

**Fig. 7:**
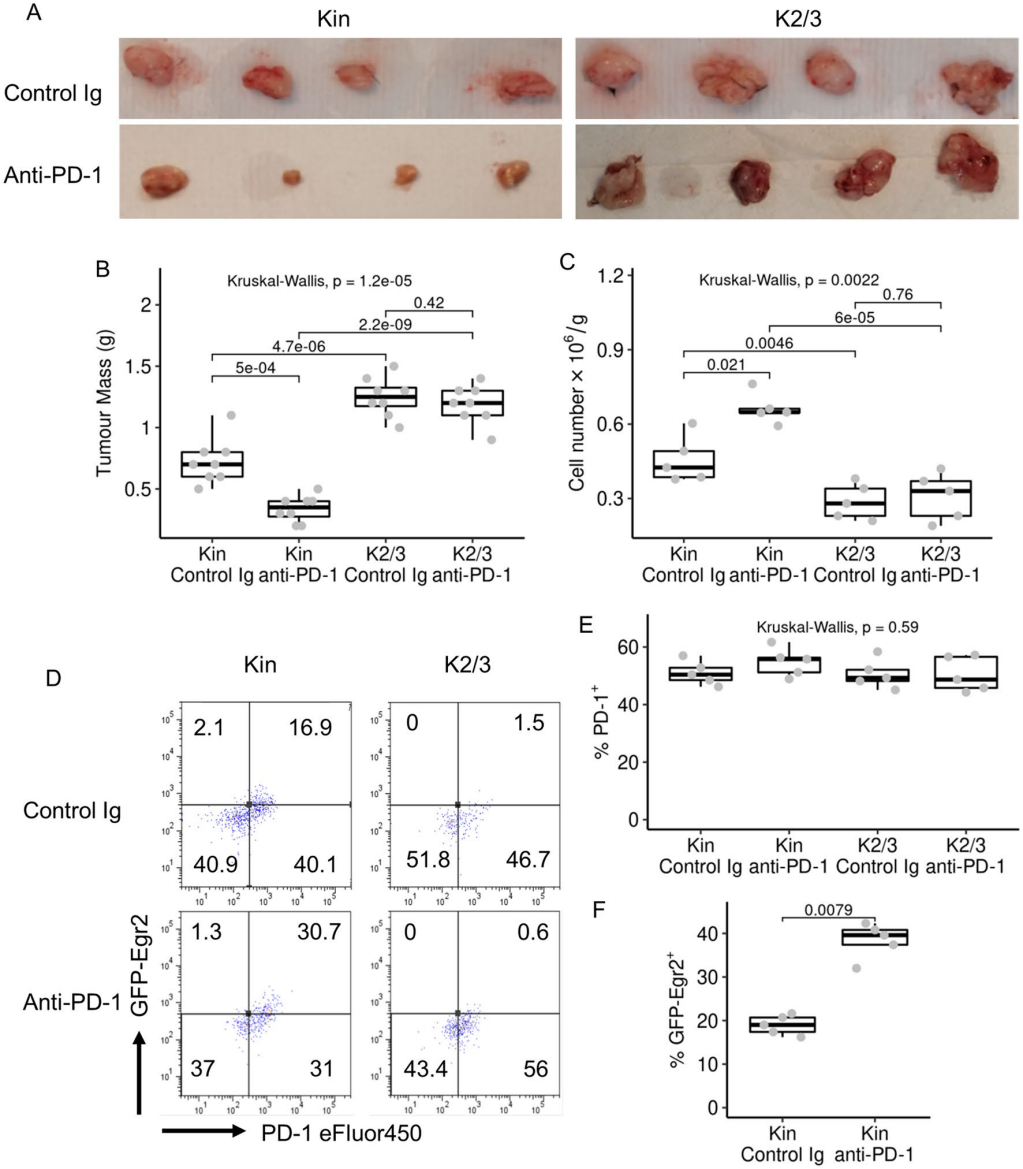
Anti-PD-1 efficacy is reduced in K2/3 mice. MC38 tumours were established in Kin (GFP-Egr2) and K2/3 (CD2-Egr2/3^-/-^) mice. Anti-PD-1 was injected I.P. every 3 days post tumour implantation. 14 days after tumour implantation, tumour mass (**A** and **B**), CD8 TIL numbers (**C**) and PD-1 (**D** and **E**) and GFP-Egr2 (**D** and **F**) expression by CD8 + TILs was analysed. Data in A, D are representative of 8 (A) or 5 (**D**) mice in each group and are representative of two independent experiments. In B, C and E, the median, upper and lower quartiles from eight (**B**) or five (**C**, **E** and **F**) mice are shown, and data were analysed with Kruskal–Wallis tests, followed by two-tailed Conover tests with Benjamini–Hochberg correction (**B**, **C** and **E**), or two-tailed Mann–Whitney tests (**F**)

Immune checkpoint blockade can be an effective immune therapy against cancers [3, 24]. To assess if Egr2/3 are important for anti-PD-1 immune therapy, we treated MC38 inoculated mice with anti-PD-1. Tumour growth in anti-PD-1 treated GFP-Egr2 mice was reduced compared to the Ig control group (Fig. 7A, B), consistent with previous reports [25, 26]. Anti-PD-1 treatment also increased the number of CD8 + TILs/g in GFP-Egr2 mice (Fig. 7C). However, anti-PD-1 treatment had no effect on tumour growth or TIL numbers in CD2-Egr2/3^-/-^ mice (Fig. 7A–C). We found that anti-PD-1 did not change the composition of PD-1 + and PD-1- CD8 + cells within TILs, but increased the percentage of Egr2high CD8 + TILs (Fig. 7D–F). Since Egr2 is important for TIL expansion (Figs. 3C, 5C, D, G and H), this increase in Egr2high cells may explain the impact of anti-PD-1 treatment on expansion of TILs in GFP-Egr2 knockin mice and also the ineffectiveness of anti-PD-1 in CD2-Egr2/3^-/-^ mice. These results suggest that Egr2 and/ or 3 are important for TIL responses to anti-PD-1 blockade.

## Discussion

Antigen persistence in chronic pathogenic conditions such as chronic viral infection and cancer can induce expression of immune checkpoint molecules on antigen specific CD8 T cells and an exhausted gene expression programme leading to dysfunction [4, 27]. Despite their impaired function, exhausted T cells are highly heterogeneous in function and are essential for the control of chronic infection and perhaps also cancer [1, 4, 28], suggesting that exhausted T cells are an important part of adaptive immunity under these conditions. Although some molecules such as TCF-1 have been found to be associated with early stage T cell exhaustion [22, 23], molecules that are important to maintain the function of exhausted T cells are largely unknown. In this study, we have discovered a unique function of Egr2/3 in maintaining anti-cancer responses of exhausted TILs. Egr2 was highly induced in a proportion of CD8 + TILs in solid tumours from cancer patients and the gene sets enriched in Egr2high CD8 + TILs were associated with activation of T cells indicating that Egr2high TILs may have enhanced effector function compared to Egr2low TILs. In tumour models, Egr2high CD8 + TILs expressed high levels of PD-1, Tim3 and Lag3, but maintained their proliferative ability and functionality. These functions are associated with genes that support metabolism (Hk2, Cad, Bcat1, Scd2, Slc7a1, Tfrc) and expansion (Myb, Plk4, Cdc45, Cks1b, Spc24) which are reduced in Egr2/3^-/-^ TILs. We further discovered that anti-PD-1 increased the proportion of Egr2high TILs, while Egr2/3^-/-^ TILs failed to respond to PD-1 blockade. Thus, Egr2 and/or 3 are important for exhausted TILs to maintain anti-tumour immune responses.

Egr2 and/or 3 have often been detected in exhausted T cells that express high levels of checkpoint molecules in chronic infection and tumours from both animal models and human patients [5–8]. We have now shown that both Egr2high and Egr2/3^-/-^ TILs express high levels of checkpoint regulators, indicating that Egr2 and/or 3 are not required for the expression of checkpoint regulators in TILs. Interestingly, although immune checkpoint regulators were more highly expressed in Egr2high than Egr2low CD8 + TILs, Egr2high cells displayed better proliferation than Egr2low TILs in response to TCR stimulation, suggesting that Egr2high exhausted CD8 + TILs still maintain functionality. Egr2 and 3 deficiency impaired the proliferation and survival of TILs but had minimal impact on effector function. Consistent with this, transcriptomic pathway analysis indicated an enrichment of Myc, E2F and G2M pathways in Egr2high TILs compared to Egr2/3^-/-^ TILs. In cancer patients, we found that TCF7, a signature gene for stem cell like exhausted T cells [22, 23], was more highly expressed in PBMCs than in tumour cells, whereas Egr2 expression was specifically associated with the TME. Tcf7 expression was not reduced in Egr2/3-/- TILs from tumour models indicating that Egr2, specifically induced in TILs, has a distinct function in maintaining the function of exhausted T cells.

Egr2 and 3 play important overlapping roles in maintaining survival and promoting antigen mediated proliferation of memory phenotype and effector T cells while controlling their inflammatory function [9, 11, 13]. However, we found that the effector function of CD8 + TILs was largely unaffected by Egr2 and 3 deficiency. Indeed, gene expression analysis of CD8 + TILs failed to give a clear indication regarding the impact of Egr2 and 3 on effector function, given that Ifng was higher in Egr2high cells whereas many other effector molecules were higher in Egr2/3^-/-^ cells. In contrast, we have now shown that Egr2 and 3 are important for the proliferation and survival of CD8 + TILs. Tumour control is impaired in CD2-Egr2/3^-/-^ mice. Thus, these data support the notion that proliferation and survival of CD8 + TILs are essential for anti-tumour responses.

CD8 TILs are key players in anti-tumour immunity and are essential for tumour control and response to anti-PD-1 therapy in the MC38 model [29]. We found that anti-PD-1 treatment reduced tumour growth and increased TIL numbers in GFP-Egr2 knockin mice but had minimal effect on tumour growth in CD2-Egr2/3^-/-^ mice. In addition to the increase of TILs, we now found that Egr2 expression is increased by anti-PD-1 treatment. Checkpoint blockade increases TIL density in tumours [30, 31] and we have demonstrated that Egr2 and 3 promote TIL proliferation. Therefore, by supporting proliferation, Egr2 may be important for TILs to respond to checkpoint blockade. This raises the intriguing possibility that Egr2 might be useful as a marker of checkpoint blockade treatment efficacy.

Despite their exhausted state, the enrichment of T cells in tumours is associated with better clinical outcome [1, 2, 30, 31]. The efficacy of immune checkpoint blockade in immune therapy of cancer further indicates that exhausted T cells are an important reservoir of anti-cancer immune T cells. However, the mechanisms that induce exhaustion and maintain adaptive immunity of exhausted T cells in tumours are largely unknown. We have demonstrated that Egr2/3, expressed in a proportion of exhausted T cells, are essential for anti-tumour immune responses and anti-PD-1 antibody increases Egr2 positive TILs. Thus, Egr2/3 might be useful as a marker of anti-tumour immune responses and strategies to enhance Egr2 expression in T cells may help to promote anti-tumour immune responses.

## Methods

### Mice

GFP-Egr2 (RRID:IMSR_EM:11,391) and CD2-Egr2/3^-/-^ (RRID:IMSR_EM:11,389) mice were reported previously [9, 11]. All mice analysed were 6–8 weeks of age unless otherwise stated. No animal was excluded from the analysis, and the number of mice used was consistent with previous experiments using similar experimental designs. All mice were maintained in the Biological Services Unit, Brunel University, and used according to established institutional guidelines under the authority of a UK Home Office project licence. The protocols and procedures used on mice were reviewed and approved by the Ethical review committee of Brunel University. Experiments were performed in accordance with UK Home Office regulations (Guidance on the Operation of Animals (Scientific Procedures) Act 1986 Amendment Regulations (SI 2012/3039)).

### Tumour cell lines

B16 and MC38 murine cancer cell lines were maintained by serial in vitro passages in RPMI 1640 supplemented with 10% heat-inactivated FCS, 0.1 mM nonessential amino acids, 1 mM sodium pyruvate, 2 mM fresh l-glutamine, 100 mg/ml streptomycin, 100 U/ml penicillin, 50 mg/ml gentamycin, 0.5 mg/ml fungizone (all from Life Technologies, Rockville, MD), and 0.05 mM 2-ME (Sigma-Aldrich, St. Louis, MO).

### In vivo tumour model

A total of 1 × 10^5^ MC38 or B16 tumour cells were injected subcutaneously (s.c.) into Kin and K2/3 mice. For PD-1 treatment, three days after tumour cell injection, mice were injected intraperitoneally (i.p.) with 200 µg of InVivoPlus rat IgG2a isotype control (anti-trinitrophenol, clone 2A3, BioXCell) (cIg) or InVivoPlus rat anti-murine PD-1 (clone RMP1-14, BioXCell) monoclonal antibody. The treatment was repeated on days 6 and 9 post tumour cell inoculation.

Mice were euthanized at day 14 after tumour injection or when the tumour reached a size of 10 mm^2^. Tumours and splenocytes were harvested for in vitro assays.

### Mouse TIL isolation

Tumour cell suspensions were prepared from solid tumours by enzymatic digestion in HBSS (Life Technologies) containing 1 mg/ml collagenase, 0.1 mg/ml DNAse I, and 2.5 U/ml of hyaluronidase (all from Sigma-Aldrich) with constant stirring for 45 min at 37 °C. Cells were then passed through a 70-µm cell strainer, washed once with HBSS, and labelled with anti-CD8 microbeads and isolated on a MACS system according to the manufacturers’ instructions (Miltenyi Biotec). For RNA-seq experiments, GFP-Egr2highCD3 + CD8 + CD44high and Egr2^-/-^Egr3^-/-^CD3 + CD8 + CD44high TILs were isolated by FACS. Where indicated, isolated TILs were labelled with Cell Trace Violet (Life Technologies) and stimulated in vitro for 72 h with 5µg/ml anti-CD3 and 2µg/ml anti-CD28. After stimulation, TILs were collected and their proliferation analysed.

### Antibodies and flow cytometry

PerCP-Cy5.5 conjugated antibody to CD3 (cat 45–0031-82, clone 145-2C11); APC or APC-eFluor780-anti-CD8 (cat 17–0081-82, 47–0081-82, clone 53–6.7); eFluor450-anti-CD279 (PD-1) antibody (cat 48–9985-82, clone J43), PE-Cy7 anti-IFNγ (cat 25–7311-82, clone XMG1.2); PE-anti-Ki-67 (cat 12–5698-82, clone SolA15), APC-eFluor780 labelled antibody to CD44 (cat 47–0441-82, clone IM7); and PE-Tim3 (cat 12–5870-82, clone RMT3-23) were obtained from eBioscience. PE-anti-mouse CD223 (LAG-3) antibody (cat 125,208 clone C9B7W), APC Annexin V (cat 640,920), and Propidium iodide (PI, cat 421,301) were from Biolegend. For analysis of IFNγ producing cells, the cells were stimulated with 50 ng/ml PMA and 200 ng/ml ionomycin in the presence of Golgistop (BD Biosciences) for 3 h and then fixed with the Foxp3 staining kit (eBioscience) and analysed by flow cytometry. For all flow cytometry analyses, data were acquired on a LSR II or Canto II (BD Immunocytometry Systems) and analysed using FlowJo (Tree Star).

### Single cell RNAseq analysis

For scRNAseq analysis, the count data for the GSE108989, GSE98638, GSE140228, and GSE99254 datasets [18–21] were downloaded from GEO. The data were read into R and CD8 T cells selected based upon the authors’ provided annotation. Next, the scran, scater and scuttle packages [32] were used to perform cell quality control filtering, scaling normalization and log transformation. For EGR2, a cutoff of 5 log normalized expression was selected to identify EGR2high cells and log normalized expression levels plotted using the ggplot2 package [33].

Dataset GSE108989 was selected for pseudobulk analysis due to the high expression of EGR2. Pseudobulk samples for EGR2 high (EGR2 > 5 as described above) and EGR2 low (EGR2 <  = 5) were generated for each patient from raw counts using the scuttle function aggregateAcrossCells. Multidimensional scaling was performed using the edgeR and limma packages [34, 35] and samples from patients with 10 or fewer EGR2 high cells were identified as outliers and discarded. EGR high and EGR2 low tumour samples were then analysed using DESeq2 [36]. Briefly, count data were first normalized and dispersion estimated using the locfit package before a negative binomial model was fitted, with patient as the reduced model, and significance assessed by a likelihood ratio test. Log fold changes were shrunk, and s-values calculated using the apeglm method. The msigdbr package was used to retrieve the annotation for the Broad Institute Hallmark gene sets [37] for use in gene set testing. Pre-ranked GSEA was performed using the clusterProfiler package [38] using the negative log10 of the s-value multiplied by the sign of the log2 fold change as the ranking metric. Normalized enrichment scores and p-values were plotted using the ggplot2 package [33].

### RNA-seq analysis

RNA was isolated and purified using TRIzol reagent (Life Technologies). RNA concentration and integrity were assessed using Qubit with a RNA HS reagent kit (ThermoScientific) and an Agilent 4200 Tapestation (Agilent Technologies), respectively. cDNA libraries were generated from independent biological replicate RNA samples using the NEBNext® Single Cell/Low Input RNA Library Prep Kit for Illumina (New England Biolabs, E6420). 200 pg of amplified cDNA was then tagmented using the Nextera XT protocol (Illumina, FC-131–1024) and amplified with custom primers containing P5/P7 sequences along with a 6 bp sample specific index. We performed 43 bp paired-end sequencing using an Illumina NextSeq 500 platform. Run data were demultiplexed and converted to fastq files using Illumina’s bcl2fastq Conversion Software v2.20. The short sequenced reads were mapped to the mm10 build of the mouse reference genome using the spliced aligner Hisat2 [39]. Intermediate processing steps to remove secondary alignments and pairs where only one read was mapped were performed using Samtools [40], while optical duplicates were removed with Picard [41]. We used several R/Bioconductor packages to identify genes differentially expressed between GFP-Egr2high and Egr2/3^−/−^ TILs. Briefly, the number of reads mapped to each gene based on the NCBI RefSeq database were counted using the featureCounts function in the Rsubread package [42] and genes differentially expressed between groups were identified using the R/Bioconductor package DESeq2 [36]. Count data were first normalized and dispersion estimated using the locfit package before a negative binomial model was fitted with significance assessed by a likelihood ratio test. Log fold changes were shrunk, and s-values calculated using the apeglm method. Genes with an s-value less than or equal to 0.05 and an absolute fold change greater than or equal to 1.5 were considered differentially expressed.

For the heatmap, a variance stabilizing transformation from the DESeq2 and vsn packages [36, 43] was applied to the dataset and genes differentially expressed between GFP-Egr2high and Egr2/3^−/−^ TILs were selected. Z-scores were calculated for each gene before hierarchical clustering using 1 – Pearson correlation as a distance metric and visualization with the ComplexHeatmap package [44].

For functional annotation, the msigdbr package was used to identify genes corresponding to the Broad Institute Hallmark gene sets [37]. Pre-ranked GSEA was performed using the clusterProfiler and fgsea R packages [38] with the negative log10 of the s-value multiplied by the sign of the log2 fold change as the ranking metric. Normalized enrichment scores and p-values were plotted using the ggplot2 package [33].

### Statistics

To analyse the statistical significance of differences between groups, two-tailed Mann–Whitney tests using the R package coin [45], or Kruskal–Wallis tests followed by pairwise comparisons using Conover tests, as implemented in the R package PMCMRplus [46], with Benjamini–Hochberg correction for multiple comparisons were used as indicated. Differences with a *p* value < 0.05 were considered significant. Box and whisker plots showing the median, first and third quartiles and whiskers extending at most 1.5 times the interquartile range were plotted using the R package ggplot2 [33].

### Supplementary Information

Below is the link to the electronic supplementary material.

Supplementary file1 (DOCX 12 KB)

Supplementary file2 (PDF 206 KB)

Supplementary file3 (XLSX 223 KB)

Supplementary file4 (XLSX 314 KB)

## Data Availability

The RNA-seq data generated in this study are available from the ArrayExpress website (www.ebi.ac.uk/arrayexpress) under accession number: E-MTAB-11672.
