# Supplementary material for: Egr2 and 3 maintain anti-tumour responses of exhausted tumour infiltrating CD8 + T cells
Source: Cancer Immunol Immunother. 2022 Nov 7;72(5):1139–51. doi: 10.1007/s00262-022-03319-w (PMC10110685; doi:10.1007/s00262-022-03319-w)
Supplement: Supplementary file 1 — Supplementary file1 (DOCX 12 KB) [file 262_2022_3319_MOESM1_ESM.docx]

Supplementary information

Supplementary information includes two supplementary tables, three supplementary figures and two supplementary data files. The two supplementary data files contain lists of genes differentially expressed between GFP-Egr2+ and Egr2/3^−/−^ TILs in the MC38 and B16 models.
