# Supplementary material for: Egr2 and 3 maintain anti-tumour responses of exhausted tumour infiltrating CD8 + T cells
Source: Cancer Immunol Immunother. 2022 Nov 7;72(5):1139–51. doi: 10.1007/s00262-022-03319-w (PMC10110685; doi:10.1007/s00262-022-03319-w)
Supplement: Supplementary file 2 — Supplementary file2 (PDF 206 KB) [file 262_2022_3319_MOESM2_ESM.pdf]

Supplementary Table 1 - proportion of CD8+ PBMCs and CD8+ TILs expressing EGR2

Cancer	sampleType	EGR2_positive	Total	EGR2_positive_percentage
CRC_GSE108989	PBMC	64	1021	6
CRC_GSE108989	Tumour	506	1646	31
HCC_GSE98638	PBMC	16	488	3
HCC_GSE98638	Tumour	88	670	13
HCC_GSE140228	PBMC	9	255	4
HCC_GSE140228	Tumour	83	476	17
NSCLC_GSE99254	PBMC	23	1077	2
NSCLC_GSE99254	Tumour	171	1956	9

Supplementary Table 2 - proportion of CD8+ TILs expressing EGR2 in individual patients from dataset CRC_GSE108989

Patient_ID	EGR2_positive	Total	EGR2_positive_percentage
P0123	29	88	33
P0215	21	109	19
P0309	23	100	23
P0411	20	82	24
P0413	44	114	39
P0701	29	135	21
P0825	64	167	38
P0909	17	122	14
P1012	80	232	34
P1207	8	78	10
P1212	77	203	38
P1228	94	216	44

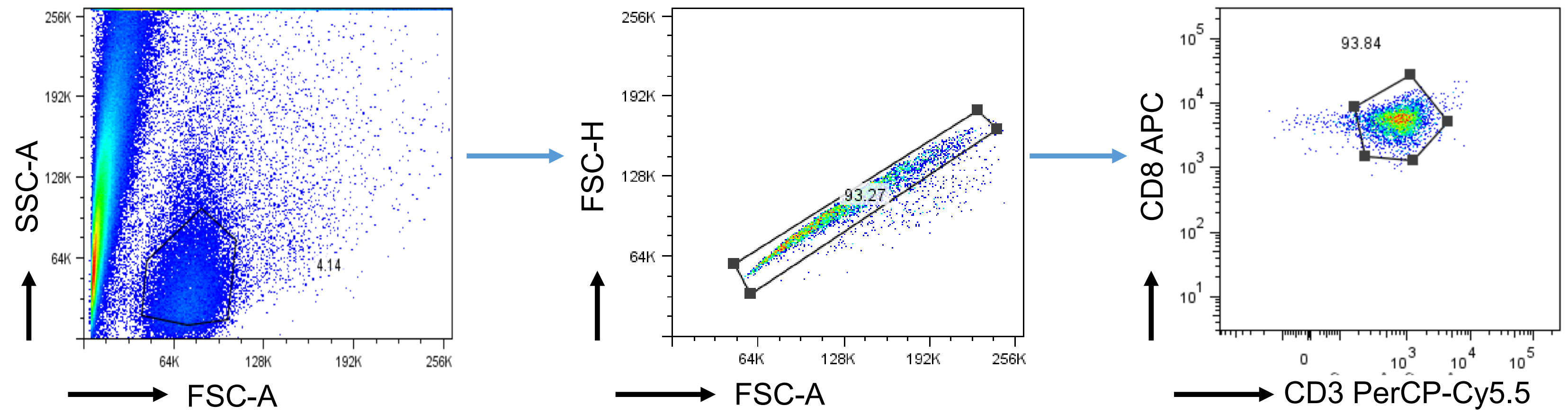

Supplementary Figure 1. Gating strategy for analysis of TILs.
CD8 TILs were isolated by MACS as described in the methods section
and then stained with fluorochrome conjugated antibodies and analysed by flow cytometry

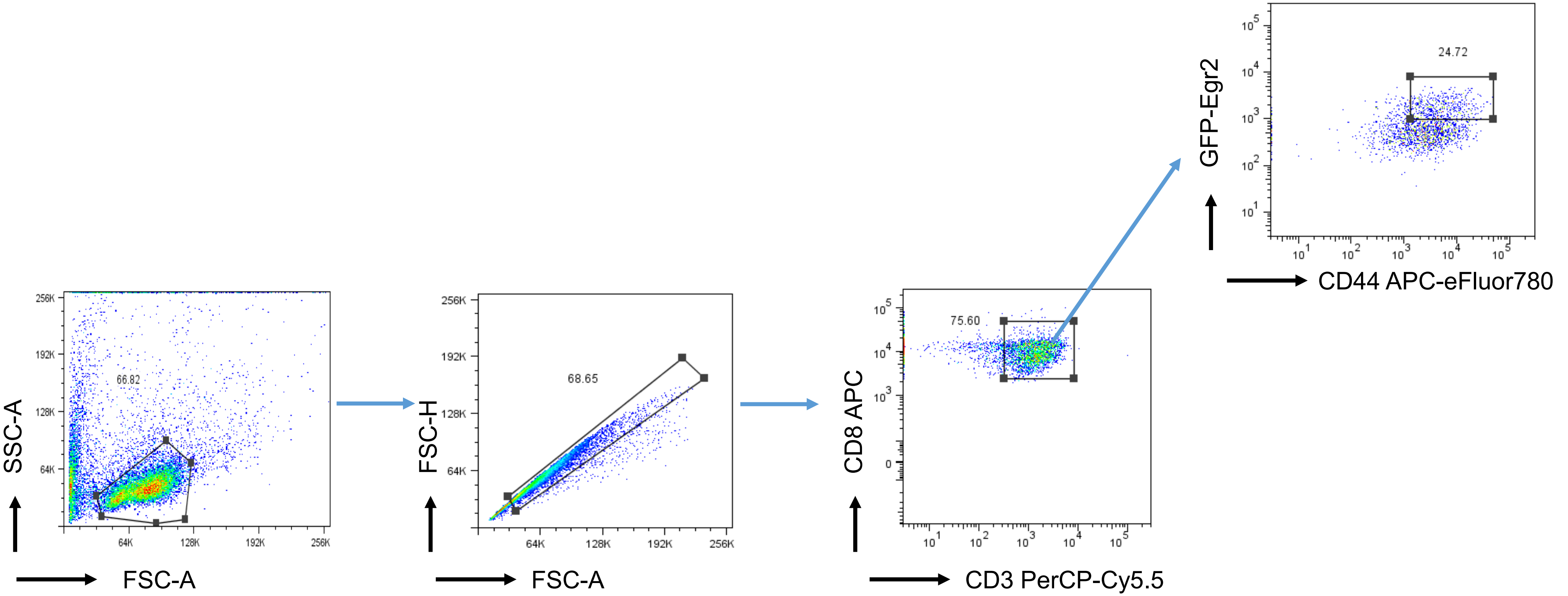

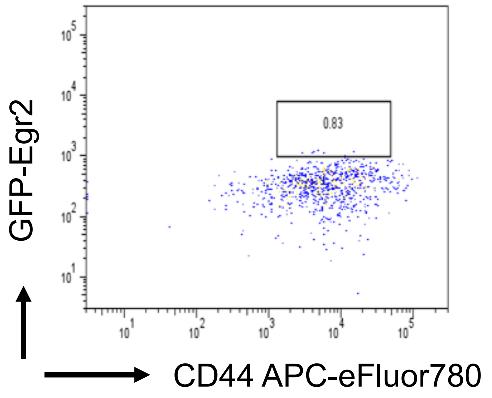

Supplementary Figure 2. Gating strategy for cell sorting.
CD8 TILs were isolated by MACS as described in the methods section and then stained with fluorochrome conjugated antibodies.
GFP-Egr2^{high}CD44high and Egr2$^{-/-}$3$^{-/-}$CD44^{high} cells were sorted by FACS and used for *in vitro* functional analysis and RNAseq

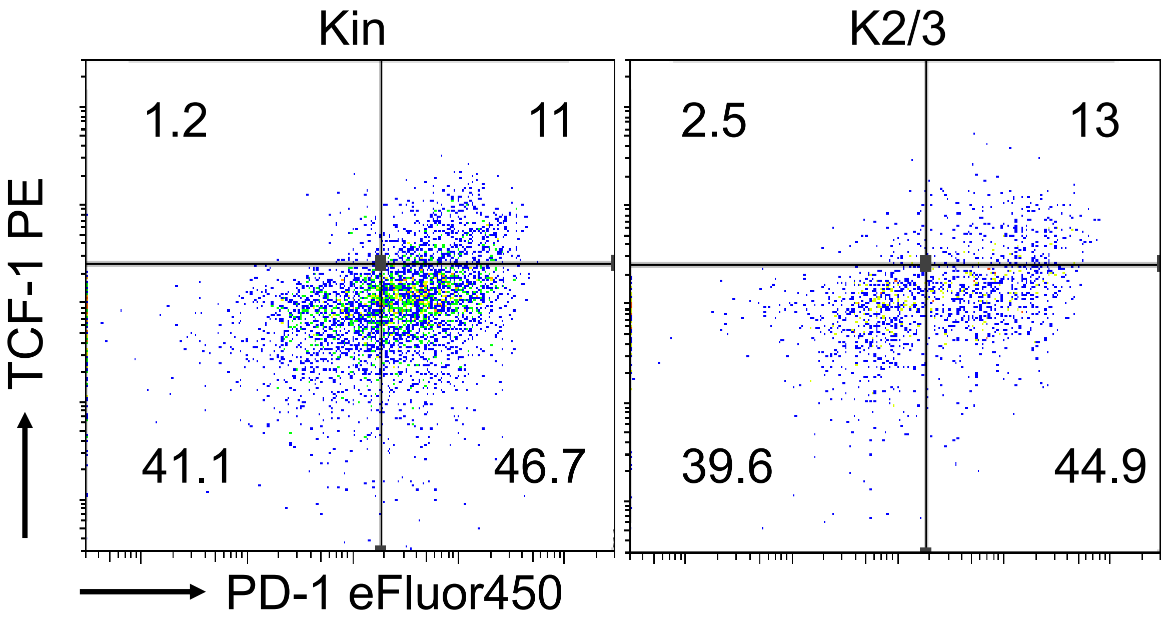

Supplementary Figure 3. Analysis of TCF-1 expressing TILs.
CD8 TILs were isolated from B16 tumours by MACS and analysed by flow cytometry
CD8+ cells were gated for analysis of TCF-1 and PD-1 expression.
Data are representative of three independent experiments.
